# A Study for Improving Compressive Strength of Cementitious Mortar Utilizing Magnetic Water

**DOI:** 10.3390/ma13081971

**Published:** 2020-04-23

**Authors:** Omar M. M. Yousry, Metwally A. Abdallah, Mariam F. Ghazy, Mohamed H. Taman, Mosbeh R. Kaloop

**Affiliations:** 1 Structural Engineering Department, Tanta University, Tanta 31733, Egypt; omr_elsheikh@f-eng.tanta.edu.eg (O.M.M.Y.); metwali.abdelatty@f-eng.tanta.edu.eg (M.A.A.); mariam.ghazy@f-eng.tanta.edu.eg (M.F.G.); mohamed.taman@f-eng.tanta.edu.eg (M.H.T.); 2 Civil and Environmental Engineering Department, Incheon National University, Incheon 22012, Korea; 3 Public Works and Civil Engineering Department, Mansoura University, Mansoura 35516, Egypt

**Keywords:** magnetic water, fly ash, superplasticizer, cementitious mortar, compressive strength, mortar flow

## Abstract

This research investigates the means to improve the compressive strength of mortar mixtures through using novel mixtures. These mixtures include magnetic water (MW) and fly ash (FA). MW was obtained by circulating tap water (TW) through a magnetic field. The magnetization duration was represented by the number of cycles, the content of FA was replaced with cement, and the super plasticizer percentage (SP) and the curing age were used and evaluated experimentally for producing the mortar mixtures. Mortar flow, crushing compressive strength, and ultrasonic pulse velocity (UPV) tests were applied to evaluate the performances of mixing characteristics. The results demonstrate that the MW-treated mortar mixtures show higher compression strength results than those prepared by TW. The compressive strength was increased up to 60% with 150 cycles, a dose of 0.5% of SP and no FA content at the age of 56 days. The dose of SP can be cut down by a maximum of 40% to 50% in cementitious mortar. the workability was enhanced by a percentage of 70%.

## 1. Introduction

Cementitious mortar is considered an important material in the construction field, as it is mainly used in concrete. However, there are some secondary uses in the activities of laying bricks, plastering, rehabilitation, and facades, which means that it has received significant attention in research [1]. A number of researches have focused on improving its properties, of which the compressive strength is the most significant [2]. Since the cement paste is the main factor in the formation of adhesives, it was necessary to pay a lot of attention to it. It is capable of reacting with water and many methods were used to increase its ability to interact and form adhesive materials [3]. One of these methods was the use of pozzolanic materials as they play a significant role in producing concrete with higher performance, greater durability, and better environmental impact [4,5]. Some pozzolanic materials such as fly ash (FA), limestone, ground blast-furnace slag, and silica fume developed and improved the efficiency of concrete properties through enhancing the paste properties [6,7].

Water, the other component that participates in the cement mixture reaction, largely controls the properties of the resulting paste according to the proportions of the salts and impurities dissolved in the water. Therefore, the researchers exerted a great effort in order to improve water quality and to set standards for its use through various means [8]. Water is a polar substance whose molecules are bound together by hydrogen bonds to form clusters [9]. If the water passes through a permanent magnetic field or an electromagnetic field, then the water is magnetized for what is known as magnetic water (MW) or magnetic field treated water (MFTW) [10].

**Figure 1:**
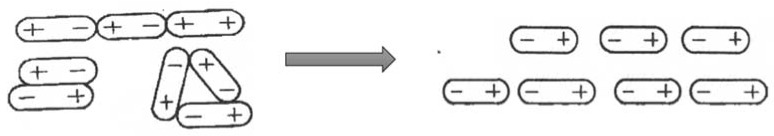
Effect of the magnetic field in water molecules [10].

MW does not mean that it has a magnetic force that can attract things. However, it relies on the magnetic field that breaks the hydrogen bonds between the water bodies that reduces the water molecular conglomerates and assists in the spread of water molecules, as shown in Figure 1 [3,10]. The magnetic field reduces the surface tension of water, which can be measured by a device called a tensiometer, and it also changes the structural composition, the viscosity, and the electrical conductivity of the water [11,12]. This, in turn, leads to a change in the path of the water molecules to be much better in the bonding between the water and any additives that can interact [13]. Consequently, this leads to a faster and more complete reaction with the cement granules when added to the water [14].

Magnetized water is very different from tap water (TW) in its mechanical, electromagnetic, and thermodynamic properties, which allows it to be used in many fields [15]. The use of magnetized water dates back to 1962 when some scientists from the Soviet Union made the first experiment on magnetized water to verify the use of magnetized water in the manufacture of special concrete blocks for installations of the armed forces, such as airports [16]. As mentioned before, when water passes through the magnetic field, it breaks down groups of water molecules so that they more easily penetrate cement particles, which increases their effective hydration and improves their susceptibility to concrete, and thus, improves concrete mechanical properties [14,17,18,19].

Su and Wu [10] demonstrated that the use of magnetized water in the mixture would enhance the workability of the concrete action by up to 30% relative to the mixtures corresponding to the same water-to-cement ratios (W/C). They studied the effects of MW produced with a different magnetic field density (MFI) (0.2, 0.4, 0.6, 0.8, 1.2, 1.35 Tesla) on compressive strength in mortar and concrete samples containing FA. The results indicated that the magnetized water under the magnetic field 0.8 and 1.2 tons had the greatest contribution (15%–20%, respectively) in enhancing the compressive strength [10]. An increase of 10% to 25% in the compressive strength of slag-containing slag has been reported as a result of using MW instead of TW [14].

Soto-Bernal et al. [20] used scanning electron microscopy(SEM) tests to demonstrate the increase of magnetic field effects in denser moist C-S-H gels, which in turn increased the compressive strength in the concrete and mortar. They also found that the water temperature increased due to the use of MW. They also established a direct relationship between the density of the magnetic field and the time of cement setting [20]. Wei et al. [21] showed that the water magnetized into the concrete decreased shrinkage. Wang et al. [22,23] noted that a significantly higher early concrete strength could be obtained if magnetized water is used instead of TW to prepare the concrete mixture.

Leung et al. [24] indicated that when using only FA in self-compacting concrete (SCC) mixes, the water absorption is less than that of the combination of both FA and silica fume (SF). Moreover, A conclusion that a significant decrease in sorptivity was exhibited when more than 20% replacement of FA was applied [24]. The results, obtained by Jalal et al. [25], demonstrated that flowability of SCC, including FA, has been improved due to absorbed water and penetrating chloride ion of longer depth, as mentioned for SF previously. Zhao et al. [26] enhanced the initial slump flow on SCC incorporating FA and ground granulated blast-furnace slag. Utilizing FA, ground granulated blast-furnace slag in the SCC had not obvious negatively affected the flowability and stability of the fresh SCC., according to the results obtained by Poon et al. [27].

Water magnetization has few drawbacks that may hinder using it widely in pre-mixing plants and building construction. One of the main problems is that the magnetization process takes a long time to ensure the required level of magnetization [25]. From the applicational point of view, an effective system should be built from non-magnetic components because of the following: the water is magnetized according to the spontaneous formation and degeneration of colloidal groups of mineral cations in magnetic water, then the water molecules are charged and show diamagnetism (substances that are magnetized in a way opposite to the direction of the magnetic field). When they face ferromagnetic materials (materials that are magnetized in the direction of the magnetic field) for example, steel, it loses its magnetic ability to steel [11,12,28]. Another obstacle is water memory that can maintain magnetic properties up to 12 h. After that range this advantage may be lost [10,28]. In many regions, getting the device used for MW is a bit difficult, which put the application on the suspension mode.

A limited number of studies studied of the effect of MW on paste and mortar incorporating FA. It seems that the dosage of FA, the percentage of superplasticizer (SP), and the time of water through which water presents in the magnetic field in terms of the number of magnetic cycles need to be more specific. A few studies were conducted on the number of water magnetizing cycles on the cementitious mortar. Furthermore, there are no investigations on cementitious mortar containing SP (type F) as well. Moreover, to the best knowledge of the authors, no research work has been carried out to verify mortars, poured using MW, under ultrasound pulse velocity (UPV). The novelty of this research may arise also in the performed analysis of variance (ANOVA) analysis discussed later. For that, the current research is an attempt to investigate these factors for improving the compressive strength of the mortar.

## 2. Experimental

### 2.1. Materials

Cement: CEM I class (42.5 N) of chemical composition given in Table 1 was utilized throughout this research, which was obtained from a local store (Suez Cement Company, Suez, Suez Governorate, Egypt). The cement was conforming to American Society for Testing and Materials ASTM C150 Type1 [28] with a specific weight 3.15. The physical and mechanical properties are measured and reported in Table 2.

**Figure 2:**
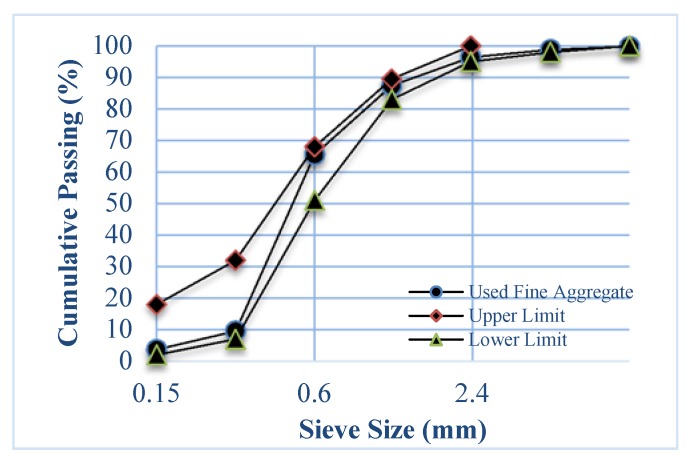
Particle size distribution (sieve analysis) for used fine aggregate.

Sand: Siliceous natural sand complying with ASTM C33 [30] was used. The sand was obtained from a local warehouse. The physical properties of the fine aggregate are given in Table 3, while the particle size distribution is given in Figure 2.

SP: Sikament 163 superplasticizer (Type F) with a specific gravity of 1.1 was produced from SIKA CO.

FA: Class (F) FA complies with the chemical of ASTM C618 and relevant international quality standards for FA with specific gravity 2.25. The chemical composition of the FA, as determined by X-ray fluorescence (XRF) analysis, is given in Table 4.

**Figure 3:**
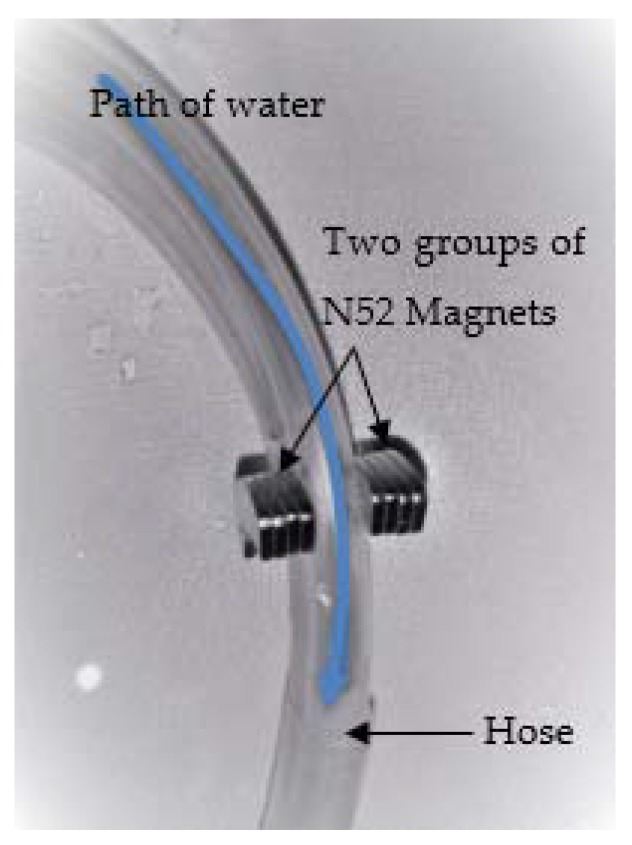
Neodymium magnetization process.

Water: two types of water were used; TW and MW. Many trials were conducted to have the water magnetized ensuring the efficiency of the magnetization process. Firstly, two huge speakers’ permanent magnets were attached facing each other. Secondly, a hose was placed through the clearance between them. Thirdly, surface tension was measured by a tensiometer and found to be 0.07157 N/m with no significant change of TW surface tension, which was 0.07199 N/ m. Fourthly, two groups of neodymium N52 magnets, as shown in Figure 3, were used following the same procedures as before. Fifthly, depending on the characteristics mentioned in the leaflet attached to it, which stated that its core magnetic intensity is 0.8 Tesla, the surface tension of the water magnetized was measured and found to be 0.07111 N/m. Finally, the decrease in surface tension result was insignificant as well.

The MW was obtained by passing water through a magnetizer with a magnetic intensity of 1.4 Tesla. The magnetizer was produced by Delta Water Company, Alexandria, Alexandria Governorate, Egypt. It was installed at the lab through some preparation to create the circulation device. This device passed the water in a cyclic way. The surface tension of water was measured and found to be 0.06624 N/m, which showed a significant change and qualified the water for the next step.

**Figure 4:**
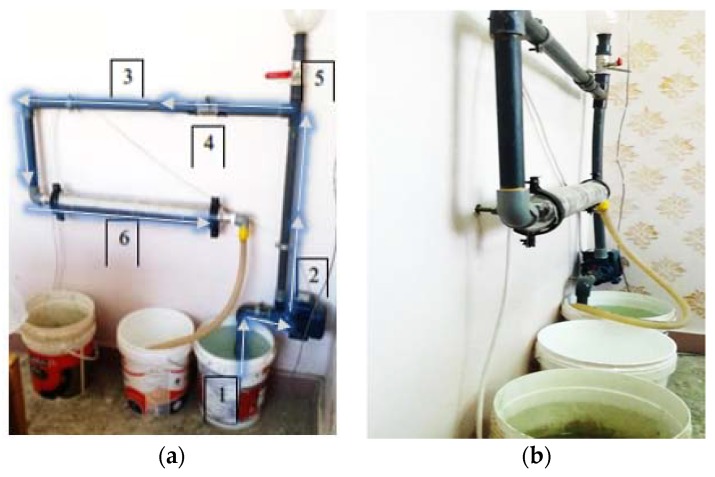
Water magnetization process. (**a**) Magnetization device composition; (**b**) Water circulation.

As shown in Figure 4a, the device consists of the following: the water source (1), this is the bucket that includes the water to be magnetized, the pump (2), which drags the water from the containing bucket then pass it upside through the pipes (3), the calibrating valve (4), A 1.5’ valve used to prepare for the pump to drag water, the water safety valve (5), the controlling valve used not to make the water returns down and to control the velocity of the passing water through the device, and the magnetizer (6), the tool itself, which carried out the magnetization operation on the water.

First, the cycle duration is determined, the pump begins pulling water up from the water source to pass it through the magnetizer and then it returns to another bucket, that is, water has achieved one cycle. The time duration required for one cycle was 36 s. According to Equation (1), the water discharge rate is calculated and found to be 9 lit/min, then stabilized using the safety valve for all mixtures.

(1) $$\mathrm{Water}\;\mathrm{discharge}\;\mathrm{Rate}\;\left(Q\right)=\frac{\mathrm{Volume}\;\left(\mathrm{lit}\right)}{\mathrm{time}\;\left(\min\right)}$$

In order to have more cycles, the time required for the targeted number of cycles is calculated by Equation (2), then the device runs for the computed time, noting that the water outlet is returned to the water source again, i.e., closed circulation as shown in Figure 4b.

(2) $$\mathrm{Targeted}\;\mathrm{Cycles}\;\mathrm{Time}\;\left(\min\right)=\;\mathrm{Number}\;\mathrm{of}\;\mathrm{cycles}\;\times \frac{\mathrm{one}\;\mathrm{cycle}\;\mathrm{time}\;\left(\sec\right)}{60\;\left(\sec/\min\right)}$$

### 2.2. Mixing Proportions

As previously mentioned, this study aimed to investigate the proper dosage of SP, the ideal dosage for the FA, and the time consumed to magnetize the water represented in the number of cycles on the cementitious mortar. The experimental program ended up with 36 mixes, which were grouped in 4 groups where each group contains 9 mixes. The water used in the first group was the TW, while the water used in the 2^nd^, 3^rd^, and 4^th^ groups was MW, differing from each other in the number of cycles, which were 50 cycles, 100 cycles, and 150 cycles on an individual basis. Hence, the effect of time consumed in magnetizing water could be investigated. Each group was split into 3 subgroups. The 1^st^ subgroup was treated with no SP, while the 2^nd^ and 3^rd^ subgroups were treated with 0.5%, 1% of SP by weight of binders, respectively. The subgroups were created in order to examine the dosage of the SP. The three mixes in each subgroup are differing from each other to investigate the effect of changing the dosage of FA by 0%, 10%, and 20% of weight of binders. The proportions of cementitious mortar mixtures are displayed in Table 5.

**Figure 5:**
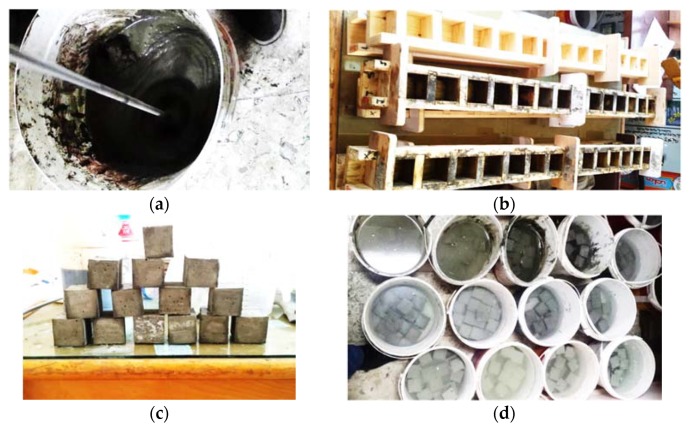
Mixing process. (**a**) Mortar mixing; (**b**) Wooden forms; (**c**) Demolded mortar samples; (**d**) Mortar specimens curing.

In order to fabricate the mortar mixes, materials to be mixed were prepared, weighted, and packed till mixing. When it comes to use the TW, the SP was added first into the water. However, when using the MW, it was put into the bucket then magnetized through the way mentioned above, then the SP was added. The duration required for 50, 100, and 150 cycles was calculated by Equation (2) to be 30, 60, and 90 min, respectively. Materials were mixed in the dry state first using a mechanical mixer in order to ensure homogeneity, then the water including the SP was added to the mixture. The full time for each mixture was 4–5 min, as can be seen in Figure 5a.

Wooden forms including separators in order to form 70 × 70 × 70 mm^3^ cubes were used for the cementitious mortar to be placed in for the compressive strength testing, as shown in Figure 5b. Wooden forms were used in order not to affect the MW [32]. After being demolded at the age of one day as can be seen Figure 5c, all specimens were cured in TW at 25 °C for 7, 28, and 56 days (Figure 5d). For all tests, triplicate specimens were used for each case study and the average of the results was then considered.

### 2.3. Testing Techniques and Procedures

#### 2.3.1. Flow Test

According to ASTM C1437-15 [33], mortar flow tests have been conducted on the 36 mortar mixtures.

#### 2.3.2. Mechanical Properties

Compressive strength test was carried out using the crushing machine of the type ELE. with maximum load 500 kN. The reported values for mortar represent the average results of three cube samples.

#### 2.3.3. Non-destructive Test

**Figure 6:**
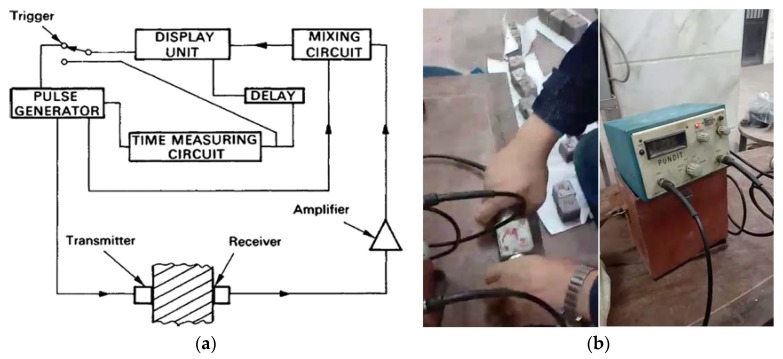
Ultrasonic pulse velocity (UPV) testing process. (**a**) UPV circuit components [35]; (**b**) UPV applying direct method.

Ultrasonic pulse velocity (UPV) test was carried out according to ASTM C597 [32]. Test equipment should provide a means to generate a pulse, transfer it through mortar, then receive and inflate the pulse computing, while showing the time spent [34]. Figure 6a shows the circuit components.

The device used was PUNDIT. In this machine, repeated pulses are generated electronically and converted to wave bursts of mechanical energy via a transmitter, which should be combined with the surface of the mortar by an appropriate medium, which was lubricant in this study. A similar receiver transformer is also coupled to the other surface of the mortar sample, i.e., 70 mm regardless of the transmitter, and mechanical energy is converted to electrical pulses of the same frequency. The electronic timing device computes the time interval between the start and reception of the pulse, and it is displayed on digital readings [35], as shown in Figure 6b.

#### 2.3.4. Statistical Analysis

As the results were huge and need somehow to be verified. Statistical analysis has been done on the result to see how significant they are. Analysis of variance (ANOVA) was performed on the results that were divided in groups.

## 3. Results and Discussion

### 3.1. Consistency in Cementitious Mortar

Table 6 shows the results of the flow test for all mixtures. There is a relative increase in flow percentages regarding the FA content, which ranges from 4.7% to 10.5% for TW mixtures. Through using MW, the relative increases in flow percentage ranges from 5.6%, 9.5%, and 8.7% to 12.4%, 21.9%, and 13% for 50-cycles, 100-cycles, and 150-cycles mixtures, respectively. Since the FA particles are smaller than cement particles, they try to fill the voids between the cement particles, which enhances the consistency of the cement mortar [10,14].

The dosage of SP plays an important role in increasing the flow ratio. The SP charged molecules attempt to disperse the cement molecules [1,2]. The last-mentioned conclusion was revealed by relative increases of flow test results. Their spans varied from 39.5%, 40.4%, 12.4%, and 10.4% to 74.4%, 69.7%, 52.4%, and 47.8%. for mixtures of 0, 50, 100, and 150 cycles, respectively. There is a followed pattern of decrease in relative increase ratios since the SP tries to equalize the charged MW molecules [21,31]. However, a slight increase of flow percentage could be observed with 0.5% dosage 50-cycle mixtures. This effect might be illustrated as 50 cycles are not enough for full magnetization, so it needs the SP.

### 3.2. Effect of Age on Cementitious Mortar

**Figure 7:**
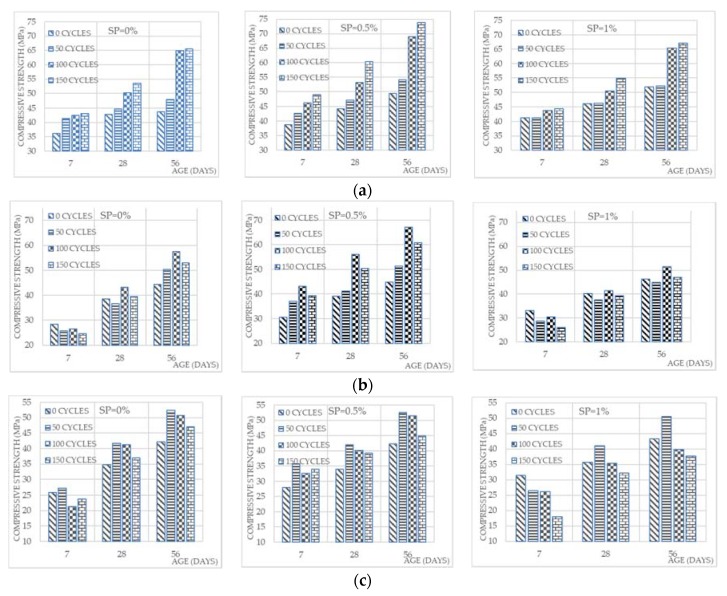
Effect of magnetic water (MW) on the compressive strength of mortar. (**a**) FA = 0.00% (**b**) FA = 10.00% (**c**) FA = 20.00%.

Figure 7 depicts the average compressive strength of mortar samples aged of 7, 28, 56 days magnetized with MW for 0, 50, 100, and 150 cycles including (a) FA = 0%, (b) FA = 10%, and (c) FA = 20%.

As shown in Figure 6, regardless of the percentage of SP and FA, the 56-day mixtures indicate the highest compression strength values. The highest value of compressive strength ever was about 70 MPa obtained by crushing the mixture treated with 150 cycles incorporating no FA content and 0.5% of SP dosage at the age of 56 days. The explanation of what happened is that mixtures are strengthened by extending the curing age [1,2,3].

When it comes to placing the samples in water and becoming wet all time, the adhesives continue to form during the reaction. MW is obviously very effective at the age of 28 and 56 days. However, it shows a slight effect at the age of 7 days. This can be discussed when MW distributes water molecules, so the reaction is carried out by cement particles and thus, it increases the compressive strength in MW mixtures more than TW mixtures [9,10,11,12,13,14,15].

Table 7 shows the relative compressive strength of mortar at ages (a) 7 days, (b) 28 days, and (c) 56 days. As noticed in Table 7, the highest increase in compressive strength at the age of 7 days is 25%, which can be observed for the mixture with no FA, a 0.5% dosage of SP and 150 cycles treatment. This might be explained as the more the water is treated, i.e., the number of cycles increased, then the higher compressive strength can be obtained. Referring to the ages of 28 and 56 days for the same mixture, the remarkable increases were 41% and 62%, respectively. The increase of compressive strength was higher than results obtained by [10] by about 20%.

### 3.3. Effect of the Number of Cycles on Cementitious Mortar

In Figure 7, the effect of the water circulating on the compressive strength follows the same trend regardless of the age. Without using FA, the best results are obtained by circulating the water for 150 cycles whatever the age was. Results showed that 150 cycles achieved the best outcomes for two reasons: First, the water was treated by MW, the higher its treatment, the greater its magnetization. Secondly: the reaction occurs between water and cement, which needs the optimum cement particle dispersion using magnetization, and for this very reason, the same number of cycles has not been reached to gain the best results in mixtures of 10% and 20% FA since the content of cement has been reduced in the mixtures. Applying this to the last-mentioned mixtures, the best compressive strengths were gained at 100 and 50 cycles, respectively.

At the age of 7 days, Table 7 reveals relative increases of 15% and 34% in the compressive strength for the ultimate 150-cycle mixture comparing it with the best 100-cycle and 50-cycle mixtures, respectively. The corresponding 28-day percentages are 10% and 30%, while at the age of 56 days they move to 14% and 40%, respectively. Therefore, the more the FA replaces the cement, the smaller number of cycles required.

### 3.4. Effect of FA Replacement on Cementitious Mortar

Despite the number of cycles used for the magnetization of water, all samples containing FA expose lower compressive strength than those without FA replacement except for ones of zero cycles. The addition of FA brought a decrease in compressive strength, and this effect has become less important because the pozzolanic reaction between FA and calcium hydroxide produced from the hydration process would be effective at later ages [10].

As shown in Figure 7, when the number of cycles is zero, i.e., using TW, there is an apparent increase, ranging from 12% to 20% in the compressive strength for the 10% FA mixtures at the ages of 28 and 56 days. This could be explained as follows: When using TW, FA starts its pozzolanic effect with a higher amount of calcium hydroxide resulting from the cement reaction, but when using MW, the cement reaction produces less calcium hydroxide complying with scanning electron microscopy Test [10], so the pozzolanic reaction occurs on a smaller scale. Thus, the compressive strength decreases. In other words, the higher the FA contents, the lower the compressive strength of the mortar treated in MW only.

### 3.5. Effect of SP Dosage on Cementitious Mortar

As shown in Figure 7, In spite of the FA-cement replacement and the number of cycles carried out, the results follow a harmonious model. Of all mixtures, 0.5% SP mixtures reveal the highest values for the MW mixes; however, 1% SP reaches the best values when using TW. This can be explained as follows: Due to the use of TW, the percentage of SP is very effective as the water particles are distributed for the reaction across the cement particles, but when using MW, which makes the same effect by rearranging the water molecules, there is no need to excess the amount of SP. This is because MW reduces the dose of SP by about 30% in concrete [36]. Therefore, MW can reduce the SP dose to about 40% to 50% in the mortar considering FA% and the number of cycles as illustrated in Table 7.

### 3.6. Effect of MW on the UPV Test

**Figure 8:**
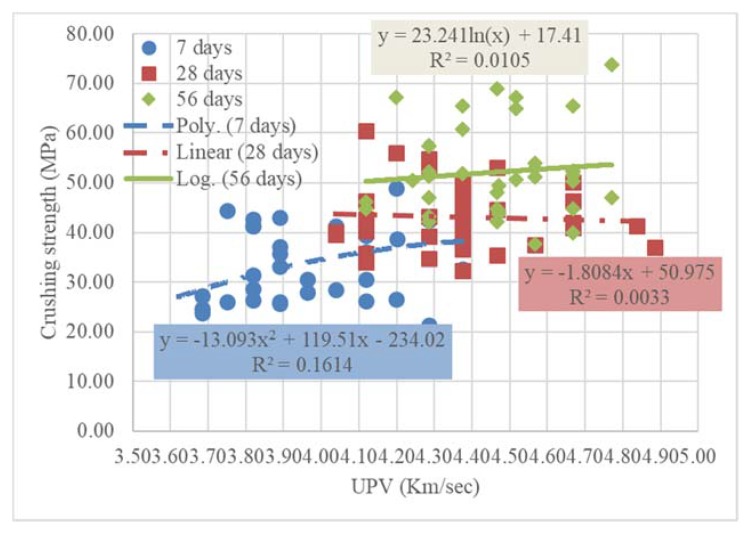
Effect of MW on ultrasonic pulse velocity (UPV) test results.

Figure 8 shows the results of the UPV values vs. the crunching compressive strength for 7, 28, and 56 days. In fact, the UPV results do not show a trend that could be followed or relied on as there was a slight difference in the results of the speeds compared to the large and wide difference in the values of the crushing strengths.

Although the velocities results were scattered as there was no pattern to trace, trend functions were deducted. The coefficient of determination, R^2^, values was so small; however, the 56-day trendline was higher than 28-day and 7-day trendlines, as shown in Figure 7. This could be explained as mentioned above, the more the curing age, the more the density of the mixture [1,2,3].

### 3.7. ANOVA Analysis

As mentioned before, the ANOVA, an extension of t-test, is a way to know how different the means of multiple groups are. To perform the test, two hypotheses are assumed. The null hypothesis states that “there is no difference between means”, so the variables do not affect the dependent variable; however, the alternative hypothesis states there is a great difference in the groups’ means, so there is one variable at least that affects the dependent variable.

By applying ANOVA to our study, an investigation was performed on the mortar compressive strength as the dependent variable and fly ash (FA), number of cycles (NC), curing age of mortar (AG), and superplasticizer dosage (SP). The analysis examined not only the significance of each independent of the four variables on the dependent variable, but also showed the interaction influence. Moreover, the accuracy of the experimental results using the least squares method have been displayed. Considering the P-value, which is the level of significance, is less than 5%, this means with a P-value greater than 0.05, the null hypothesis should be accepted. However, when the P-value is less than 0.05, the alternative hypothesis should be accepted.

Referring to [37], all the results of ANOVA statistical analyses have been displayed in Table 8. It can be easily seen that all the results of P-value are less than 5%. Hence, the null hypothesis should not be accepted. Therefore, all the independent variables without exceptions surely affect the dependent variable (the mortar compressive strength). Also, regarding to the F-values, this evidence leads to the fact that at least one of the independent variables affects the mortar compressive strength. The model does explain a significant effect in mortar compressive strength when the combination of dependent variables was involved, particularly NC*SP*AG.

## 4. Conclusions

A study was done on the cementitious mortar incorporating FA using MW treatment for mixing the ingredients of each mixture. Thirty-six mortar mixtures were prepared to investigate the compressive strength. A magnetizing device was used to obtain the MW treated for 50, 100, and 150 cycles in order to examine the effect of the magnetizing time; however, TW was used to cure the samples for 7, 28, and 56 days. From all the above discussion, it can be concluded that:

- The use of MW enhances the workability of mortar and so does the FA by about 70% at most.
- The use of MW incorporating SP negatively affects the enhancement of workability.
- The use of MW can enhance the compressive strength of cementitious mortar that includes FA. The increasing percentage of the compressive strength relies on the number of cycles the water is magnetized with and the percentage of the FA.
- The best results were found when magnetizing water by 150 cycles with no FA used. The largest increase of the compressive strength was about 60% at the age of 56 days.
- With longer curing age, the pattern of compressive strength increase shows significant difference that reaches 40% between mortar prepared with MW and mortar prepared with TW.
- The compressive strength results of the mortar with zero content of FA is highly enhanced by a range of 12% to 20%, rather than the mortar with FA replacement.
- The optimum dosage of SP was 0.5% incorporating MW, regardless of the number of cycles required, shows the best results. Also, the SP dosage could be reduced by about 40%–50% at most to get the same compressive strength of the same mixture of cementitious mortar.
- The best number of cycles was 50, 100, and 150 cycles for mixtures with 20%, 10%, and 0% of FA, respectively. Hence, the less FA content is used, the more the number of cycles is required for magnetization.
- The UPV test values were not reliable and did not reflect the effect of MW on cementitious mortar.

## Figures and Tables

**Table 1:** Chemical composition of used cement [29].

Element	SiO_2_	Al_2_O_3_	Fe_2_O_3_	CaO	MgO	Na_2_O	SO_3_	K_2_O	LOI
Mass%	21.3	4.6	3.58	63.1	2.41	0.36	2.82	0.22	2.15
ASTM C150 Limit	−	−	−	−	≤ 6	−	≤ 3.5	−	−
Bouge Composition	C_3_S	C_2_S	C_3_A	C_4_AF					
Mass%	46	26	6	11					
Limit	−	−	−	−					

**Table 2:** Physical and mechanical properties of used cement.

Element	Measure	Unit	Condition
Blain fineness	3000	cm^2^/g	−
Initial setting time	180	min	Tap water
Final setting time	300	min	Tap Water

**Table 3:** Physical properties of used sand.

Element	Measure	Unit
Fineness modulus	3.31	−
Specific gravity (r_s_)	2.672	−
Bulk density (r’_s_)	1573.61	Kg/m^3^
moisture content	1.56	%
Volume increase	0.919	%
Water absorption	0.818	%

**Table 4:** Chemical composition of used fly ash (FA) [31].

Element	SiO_2_	Al_2_O_3_	Fe_2_O_3_	CaO	MgO	Na_2_O	SO_3_	K_2_O	TiO_2_	P_2_O_5_	LOI
Mass%	60.25	28.6	4.8	1.19	0.24	0.01	0.04	1.08	2.31	0.52	0.55

**Table 5:** Mixture proportions of mortar mixes (% of binders) incorporating (W/B=0.4).

Mix ID	CEM (I) (%)	FA (%)	SP (%)	No. of cycles	Sand (%)	Mix ID	CEM (I) (%)	FA (%)	SP (%)	No. of cycles	Sand (%)
TM-000-0.0-00	100	0	0	0	300	MM-050-0.0-00	100	0	0	50	300
TM-000-0.0-10	90	10	0	0	300	MM-050-0.0-10	90	10	0	50	300
TM-000-0.0-20	80	20	0	0	300	MM-050-0.0-20	80	20	0	50	300
TM-000-0.5-00	100	0	0.5	0	300	MM-050-0.5-00	100	0	0.5	50	300
TM-000-0.5-10	90	10	0.5	0	300	MM-050-0.5-10	90	10	0.5	50	300
TM-000-0.5-20	80	20	0.5	0	300	MM-050-0.5-20	80	20	0.5	50	300
TM-000-1.0-00	100	0	1	0	300	MM-050-1.0-00	100	0	1	50	300
TM-000-1.0-10	90	10	1	0	300	MM-050-1.0-10	90	10	1	50	300
TM-000-1.0-20	80	20	1	0	300	MM-050-1.0-20	80	20	1	50	300
MM-100-0.0-00	100	0	0	100	300	MM-150-0.0-00	100	0	0	150	300
MM-100-0.0-10	90	10	0	100	300	MM-150-0.0-10	90	10	0	150	300
MM-100-0.0-20	80	20	0	100	300	MM-150-0.0-20	80	20	0	150	300
MM-100-0.5-00	100	0	0.5	100	300	MM-150-0.5-00	100	0	0.5	150	300
MM-100-0.5-10	90	10	0.5	100	300	MM-150-0.5-10	90	10	0.5	150	300
MM-100-0.5-20	80	20	0.5	100	300	MM-150-0.5-20	80	20	0.5	150	300
MM-100-1.0-00	100	0	1	100	300	MM-150-1.0-00	100	0	1	150	300
MM-100-1.0-10	90	10	1	100	300	MM-150-1.0-10	90	10	1	150	300
MM-100-1.0-20	80	20	1	100	300	MM-150-1.0-20	80	20	1	150	300

**Table 6:** Flow ratio of mortar mixes.

Mix ID	Flow (%)	Mix ID	Flow (%)	Mix ID	Flow (%)	Mix ID	Flow (%)
TM-000-0.0-00	86%	MM-050-0.0-00	89%	MM-100-0.0-00	105%	MM-150-0.0-00	115%
TM-000-0.0-10	90%	MM-050-0.0-10	94%	MM-100-0.0-10	115%	MM-150-0.0-10	125%
TM-000-0.0-20	95%	MM-050-0.0-20	100%	MM-100-0.0-20	128%	MM-150-0.0-20	130%
TM-000-0.5-00	120%	MM-050-0.5-00	125%	MM-100-0.5-00	118%	MM-150-0.5-00	127%
TM-000-0.5-10	130%	MM-050-0.5-10	138%	MM-100-0.5-10	130%	MM-150-0.5-10	137%
TM-000-0.5-20	138%	MM-050-0.5-20	148%	MM-100-0.5-20	145%	MM-150-0.5-20	147%
TM-000-1.0-00	150%	MM-050-1.0-00	151%	MM-100-1.0-00	160%	MM-150-1.0-00	170%
TM-000-1.0-10	160%	MM-050-1.0-10	168%	MM-100-1.0-10	175%	MM-150-1.0-10	180%
TM-000-1.0-20	170%	MM-050-1.0-20	178%	MM-100-1.0-20	185%	MM-150-1.0-20	200%

**Table 7:** Relative compressive strength of mortar.

**Age (Days)**		**(a): 7**								
FA (%)		0			10			20		
SP (%)		0	0.5	1	0	0.5	1	0	0.5	1
No. of cycles	0	100%	107%	104%	73%	104%	71%	66%	86%	65%
	50	105%	96%	105%	66%	95%	73%	70%	91%	68%
	100	109%	118%	112%	68%	110%	78%	55%	83%	67%
	150	110%	125%	114%	63%	101%	67%	61%	87%	46%
**Age (Days)**		**(b): 28**								
FA (%)		0			10			20		
SP (%)		0	0.5	1	0	0.5	1	0	0.5	1
No. of cycles	0	100%	112%	108%	90%	102%	94%	81%	79%	74%
	50	104%	104%	109%	86%	101%	88%	98%	98%	96%
	100	118%	125%	118%	101%	131%	97%	97%	94%	83%
	150	125%	141%	129%	92%	118%	92%	87%	92%	76%
**Age (Days)**		**(c): 56**								
FA (%)		0			10			20		
SP (%)		0	0.5	1	0	0.5	1	0	0.5	1
No. of cycles	0	100%	117%	114%	110%	112%	98%	98%	86%	84%
	50	105%	118%	123%	93%	98%	102%	115%	115%	111%
	100	142%	151%	143%	126%	147%	113%	111%	113%	87%
	150	143%	162%	147%	116%	133%	103%	103%	98%	83%

**Table 8:** Multiple Analysis of Variances (MANOVA) for compressive strength of mortar.

ANOVA for Multiple						
Source	Sum Square	Degree of Freedom	Singular	Mean Square	F	Prob > F
FA	3179.289251	2	0	1589.644625	895.1554369	2.871E-23
NC	726.0993493	3	0	242.0331164	136.29289	3.311E-15
SP	575.234355	2	0	287.6171775	161.9620361	1.161E-14
Ag	6109.956227	2	0	3054.978114	1720.309197	1.221E-26
FA*NC	855.1556863	6	0	142.5259477	80.25874146	1.090E-14
FA*SP	172.1672051	4	0	43.04180127	24.23755713	3.957E-08
FA*AG	72.54380606	4	0	18.13595152	10.21265718	5.649E-05
NC*SP	366.8239729	6	0	61.13732881	34.4274509	1.203E-10
NC*AG	294.1447032	6	0	49.0241172	27.60629914	1.198E-09
SP*AG	77.4013099	4	0	19.35032747	10.89649257	3.513E-05
FA*NC*SP	86.70311295	12	0	7.225259412	4.068664243	1.676E-03
FA*NC*AG	173.1130465	12	0	14.42608721	8.123570636	8.017E-06
FA*SP*AG	89.67728209	8	0	11.20966026	6.312346904	1.979E-04
NC*SP*AG	53.15810426	12	0	4.429842021	2.494518024	2.734E-02
Error	42.61994	24	0	1.775830833	−	−
Total	12874.08735	107	0	−	−	−

* means combining these variables with each other.
